# Gliadel wafer implantation combined with standard radiotherapy and concurrent followed by adjuvant temozolomide for treatment of newly diagnosed high-grade glioma: a systematic literature review

**DOI:** 10.1186/s12957-016-0975-5

**Published:** 2016-08-24

**Authors:** Lynn S. Ashby, Kris A. Smith, Baldassarre Stea

**Affiliations:** 1 Department of Neurology, Barrow Neurological Institute, 500 W. Thomas Rd, Suite 300, Phoenix, AZ 85013 USA; 2 Department of Neurosurgery, Barrow Neurological Institute, Phoenix, AZ 85013 USA; 3 Department of Radiation Oncology, Arizona Cancer Center, University of Arizona, Tucson, AZ 85724 USA

**Keywords:** High-grade glioma (HGG), Glioblastoma multiforme (GBM), Gliadel wafers, Radiotherapy (RT), Temozolomide (TMZ), Systematic review, Efficacy, Safety

## Abstract

Since 2003, only two chemotherapeutic agents, evaluated in phase III trials, have been approved by the US Food and Drug Administration for treatment of newly diagnosed high-grade glioma (HGG): Gliadel wafers (intracranially implanted local chemotherapy) and temozolomide (TMZ) (systemic chemotherapy). Neither agent is curative, but each has been shown to improve median overall survival (OS) compared to radiotherapy (RT) alone. To date, no phase III trial has tested these agents when used in sequential combination; however, a number of smaller trials have reported favorable results. We performed a systematic literature review to evaluate the combination of Gliadel wafers with standard RT (60 Gy) plus concurrent and adjuvant TMZ (RT/TMZ) for newly diagnosed HGG. A literature search was conducted for the period of January 1995 to September 2015. Data were extracted and categorized, and means and ranges were determined. A total of 11 publications met criteria, three prospective trials and eight retrospective studies, representing 411 patients who received Gliadel plus standard RT/TMZ. Patients were similar in age, gender, and performance status. The weighted mean of median OS was 18.2 months (ten trials, *n* = 379, range 12.7 to 21.3 months), and the weighted mean of median progression-free survival was 9.7 months (seven trials, *n* = 287, range 7 to 12.9 months). The most commonly reported grade 3 and 4 adverse events were myelosuppression (10.22 %), neurologic deficit (7.8 %), and healing abnormalities (4.3 %). Adverse events reflected the distinct independent safety profiles of Gliadel wafers and RT/TMZ, with little evidence of enhanced toxicity from their use in sequential combination. In the 11 identified trials, an increased benefit from sequentially combining Gliadel wafers with RT/TMZ was strongly suggested. Median OS tended to be improved by 3 to 4 months beyond that observed for Gliadel wafers or TMZ when used alone in the respective phase III trials. Larger prospective trials of Gliadel plus RT/TMZ are warranted.

## Background

Gliadel wafers (Arbor Pharmaceuticals, Atlanta, GA) are biodegradable copolymers (prolifeprospan 20) impregnated with the alkylating agent carmustine (1,3-bis(2-chloroethyl)-1-nitrosurea (BCNU)). Gliadel wafers were developed for treatment of high-grade glioma (HGG) beginning in the 1990s in order to overcome the limitations of blood-brain barrier impermeability to antineoplastic agents. Despite aggressive gross total surgical resection, HGG remains incurable because of the infiltrative nature of the disease, which progresses diffusely but commonly recurs locally within 2 cm of the original tumor bed [1]. Approved by the US Food and Drug Administration (FDA) as an active antineoplastic agent administered intravenously for treatment of glioblastoma multiforme (GBM), carmustine was selected as the best candidate for development of surgically implantable polymers because of its documented efficacy [2–4].

Initial work by Brem et al. demonstrated the safety and efficacy of Gliadel wafers for GBM in humans [5–7]. A phase III multicenter, double-blind trial in 222 patients with recurrent GBM demonstrated increased median overall survival (OS) with Gliadel wafers compared with placebo wafers (31 vs 23 weeks, hazard ratio (HR) 0.67, 95 % confidence interval (CI) 0.51–0.90, *p* = 0.006) [5]. Subsequently, based on the survival advantage seen in recurrent GBM, Gliadel wafer implantation was investigated as potential initial therapy in patients with newly diagnosed HGG tumors in two phase III multicenter, double-blind, placebo-controlled trials [8–10]. Both trials reported a significant increase in median OS for patients implanted with Gliadel wafers compared to those implanted with placebo wafers. In the 32-patient Valtonen et al.’s trial of surgical resection and implantation of Gliadel wafers versus placebo, median OS increased from 39.9 to 58.1 weeks (*p* = 0.012) [9]. In the 240-patient Westphal et al.’s trial, median OS increased from 11.6 to 13.9 months (log-rank *p* = 0.03, stratified by country) [8, 10]. The FDA approved Gliadel wafers for the treatment of recurrent GBM in 1997 and for the treatment of newly diagnosed HGG (World Health Organization (WHO) grade III and grade IV glioma) in 2003.

In 2005, the European Organization for Research and Treatment of Cancer (EORTC) Brain Tumor and Radiotherapy Groups and the National Cancer Institute of Canada (NCIC) published the results of a phase III multicenter, double-blind, placebo-controlled trial treating 573 patients with radiotherapy (RT) alone versus RT with concurrent daily oral temozolomide (TMZ) followed by 6 cycles of adjuvant TMZ for five consecutive days every 28 days (a regimen abbreviated here as RT/TMZ) [11, 12]. The EORTC/NCIC study demonstrated significant improvements with RT/TMZ compared to RT alone: median OS increased from 12.1 months (95 % CI 11.2–13.0) to 14.6 months (95 % CI 13.2–16.8), and 2-year OS increased from 10.9 % (95 % CI 6.8–14.1 %) to 27.2 % (95 % CI 21.2–31.7 %). In 2005, the FDA approved TMZ (Temodar, Merck & Co., Kenilworth, NJ) for use in newly diagnosed GBM in accordance with the dose schedule established by the EORTC/NCIC study. Thereafter, this RT/TMZ regimen has been generally adopted as the “standard of care” for histologically confirmed GBM following surgical resection at initial diagnosis.

The phase III trials of Gliadel wafers and the EORTC/NCIC phase III trial of RT/TMZ represented major contributions to the progress of treatment for adults with HGG [13–15]. Nonetheless, the prognosis for newly diagnosed HGG, especially for GBM, continues to be unacceptably poor. For the period of 1999 to 2011, as reported by the Central Brain Tumor Registry of the USA, 1-year and 2-year survival rates for patients with GBM were 36.5 and 14.8 %, respectively [16]. More recent large phase III trials of approaches such as dose-intensification schedules of TMZ, the addition of the anti-angiogenic agent bevacizumab, or other novel therapies like cilengitide have failed to show improved survival for GBM beyond what was originally achieved with RT/TMZ in the EORTC/NCIC trial [17–20]. Likewise, the strategy of increasing radiation dose by either radioactive seed implantation or stereotactic radiosurgery failed to achieve meaningful increases in median OS [21, 22].

With very few FDA-approved treatments available for patients with newly diagnosed GBM, and with even fewer available for anaplastic WHO grade III glioma, sequential treatment with Gliadel wafers at the time of surgical resection followed by the RT/TMZ regimen warrants reasonable consideration. Although neither agent is curative, Gliadel wafers and TMZ have each increased median survival by about 2 months when administered with RT compared to RT alone. Further improvement in survival might result from the combination of the tumoricidal mechanisms and effects of these agents used sequentially. Moreover, between surgical resection and initiation of cranial radiation, there is a scheduled delay varying from 3 to 6 weeks—a critical time gap during which Gliadel wafers provide active antineoplastic treatment when implanted post-resection.

Concerns have been expressed about potential adverse effects when Gliadel wafers are delivered as part of a regimen with standard RT/TMZ because sequential administration of the therapies has not been evaluated in a prospective, randomized, phase III trial [23]. However, as a significant number of small phase I and II trials have been conducted globally testing this hypothesis, we felt that it would be worthwhile to analyze this body of literature in order to derive a clinically meaningful consensus opinion. In this systematic literature review, we analyze the relative risks and benefits of the multimodal combination of Gliadel wafers plus standard RT/TMZ for treatment of patients with newly diagnosed HGG.

## Methods

### Search methodology

The purpose of this systematic review is to assess and summarize the complex body of literature on the use of Gliadel plus standard RT/TMZ in the treatment of newly diagnosed HGG, resolving conflicting reports and evaluating the consistency of results in these trials.

**Fig. 1:**
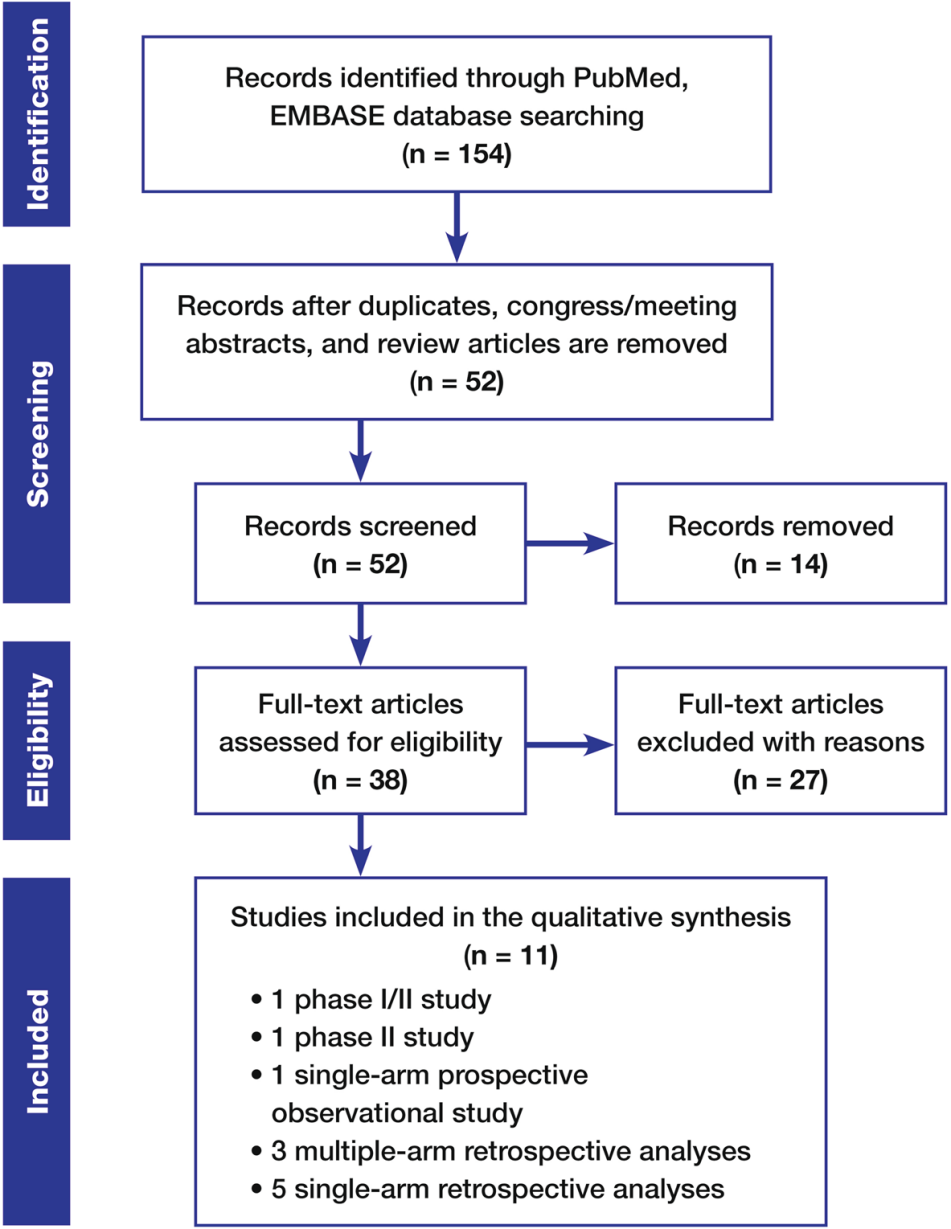
Flow diagram of the literature search for trials about Gliadel wafers combined with standard radiotherapy and concurrent followed by adjuvant temozolomide for the treatment of high-grade glioma structured in accordance with the Preferred Reporting Items for Systematic Reviews and Meta-Analyses (PRISM) schema [26]

A literature search of PubMed and EMBASE was conducted in September 2015 to identify prospective and retrospective clinical trials of Gliadel plus standard RT/TMZ (Fig. 1). The inclusive search dates were from January 1995 through September 2015. Specific search terms included Gliadel, carmustine, BCNU, wafers, and TMZ. The search results were filtered and restricted to clinical trials in humans with abstracts and full manuscripts, excluding reports that were limited to conference or congress abstracts.

After the searches were completed, the abstract of each identified publication was reviewed to determine relevance. All studies in which Gliadel wafers were implanted at the time of surgical resection and followed by RT/TMZ were obtained, and their reference lists were reviewed. Excluded from analysis were review articles, editorials, and clinical trials of therapies other than the multimodal combination therapy under investigation. Individual case reports of Gliadel plus standard RT/TMZ were also excluded. We eliminated any duplicate subject cohorts reported in more than one publication.

### Data extraction

The efficacy variables assessed were those related to survival: median OS in months, 1- and 2-year OS rates, median progression-free survival (PFS) in months, and 6-month and 1-year PFS rates.

Data on grade 3 and 4 adverse events were also extracted and reviewed. In the *Common Terminology Criteria for Adverse Events* (*CTCAE*), version 4.0, a grade 3 adverse event is defined as “severe or medically significant but not immediately life-threatening; hospitalization or prolongation of hospitalization indicated; disabling; limited self-care activities of daily living.” A grade 4 adverse event is one that has “life-threatening consequences, where urgent intervention is needed” [24]. The adverse events reported in the 11 trials included in this analysis were organized into 12 different categories: (1) cerebral edema; (2) healing abnormalities (including cerebral spinal fluid (CSF) leak and hydrocephalus); (3) intracranial hypertension; (4) intracranial infection (including abscess, cerebritis, hydrocephitis, and meningitis); (5) neurological deficit (including aphasia, change of mental status, epilepsy, and hemiparesis); (6) seizures; (7) elevated liver enzymes; (8) fatigue; (9) gastrointestinal disorders (including nausea, vomiting, and constipation); (10) myelosuppression (including anemia, lymphopenia, leukocytopenia, and thrombocytopenia); (11) skin and subcutaneous disorders; and (12) thromboembolic events (including deep vein thrombosis (DVT) and pulmonary embolism).

For comparison purposes, these adverse event data points were also extracted from the publication of the EORTC/NCIC phase III study of RT/TMZ, as well as from the two pivotal phase III clinical trials of Gliadel wafers for treatment of newly diagnosed HGG, as reported in the US product label for Gliadel wafers [8–12, 25].

## Results

### Search results

The PubMed and EMBASE literature searches yielded a combined pool of 154 possible references. Duplicates between the databases were eliminated; conference or congress abstracts were eliminated; and case reports or articles not related to the treatment of HGG were eliminated. Thirty-eight articles remained (including review articles), and these were obtained and analyzed specifically for prospective or retrospective clinical trials in which Gliadel wafers were combined with RT/TMZ for treatment of HGG. A total of 11 articles were found that reported efficacy and safety results for Gliadel plus standard RT/TMZ, without duplication of subject cohorts. A flow diagram of the literature search strategy and results, structured in accordance with the Preferred Reporting Items for Systematic Reviews and Meta-Analyses schema, appears in Fig. 1 [26].

### Study characteristics

Summarized in Table 1 are the 11 trials selected for inclusion in this analysis. The 11 reports were published between 2009 and 2015 by investigators from France (*n* = 5), Japan (*n* = 1), Germany (*n* = 1), Italy (*n* = 1), and the USA (*n* = 3) [27–37]. Five of the trials were conducted at multiple sites. Three were *prospective* clinical trials of Gliadel plus standard RT/TMZ, including one phase I/II study from Japan (which also included patients with recurrent HGG) and one phase II study from the USA. Eight were *retrospective* clinical trials.

**Table 1:** Study characteristics reported in 11 trials of Gliadel wafers combined with standard radiotherapy and concurrent followed by adjuvant temozolomide for treatment of newly diagnosed high-grade glioma

Clinical trial	Yr	Country	Sites	Study type Treatment years	Treatments/arms	ND HGG (*n*)	Gliadel wafer plus RT/TMZ (*n*)	GBM (*n*)	Other HGG (*n*)
Aoki et al. [27]	2014	Japan	10	Phase I/II study 2009 to 2012	Resection + Gliadel wafer + RT/TMZ	16	16	9	7
Bock et al. [28]	2010	Germany	7	Retrospective analysis for safety risks 2005 to 2008	Resection + Gliadel wafer + RT/TMZ	44	44	44	0
Burri et al. [29]	2015	USA	4	Phase II/2003 to 2008	Resection + Gliadel wafer + early TMZ (day 4) + RT/TMZ	46	46	43	3
Duntze et al. [30]	2012	France	17	Prospective, observational 2007 to 2009	Resection + Gliadel wafer + RT/TMZ	92	65	74	18
McGirt et al. [31]	2009	USA	1	Retrospective analysis 1997 to 2006	(1) Resection + Gliadel wafer + RT/TMZ	33	33	33	0
					(2) Resection + Gliadel wafer + RT	78		78	0
					(3) Resection/biopsy + RT/TMZ	45		45	0
Menei et al. [32]	2010	France	26	Retrospective analysis 2005 to 2006	(1) Resection + Gliadel wafer + RT/TMZ	43	43	72	11
					(2) Resection + Gliadel wafer + other regimens	40			
Miglierini et al. [33]	2012	France	1	Retrospective analysis 2006 to 2010	Resection + Gliadel wafer + RT/TMZ	24	22	16	8
Noel et al. [34]	2012	France	1	Retrospective analysis 2007 to 2008	(1) Resection + Gliadel wafer + RT/TMZ	28	28	20	8
					(2) Resection/biopsy + RT/TMZ	37		16	21
Pan et al. [35]	2008	USA	1	Retrospective analysis 2003 to 2005	Resection + Gliadel wafer + RT/TMZ	21	21	21	0
Pavlov et al. [36]	2015	France	1	Retrospective analysis 2004 to 2012	Resection + Gliadel wafer + RT/TMZ	83	61	83	0
Salvati et al. [37]	2011	Rome	1	Retrospective analysis 2006 to 2008	Resection + Gliadel wafer + RT/TMZ	32	32	NA	NA

*GBM* glioblastoma multiforme, *HGG* high-grade glioma, *ND HGG* newly diagnosed high-grade glioma, *RT/TMZ* radiotherapy + temozolomide regimen, *Yr* year published, *NA* not available

Two of the retrospective studies compared Gliadel plus standard RT/TMZ with other treatment arms. In a single-center retrospective study, McGirt et al. compared Gliadel plus RT/TMZ (*n* = 33) to treatment with Gliadel wafers alone (*n* = 78) and to a third group treated with standard RT/TMZ alone (*n* = 45) [31]. In another single-center retrospective study, Noel et al. compared Gliadel plus standard RT/TMZ (*n* = 28) with standard RT/TMZ alone (*n* = 45) [34].

In one of the 11 trials, begun before the EORTC/NCIC results had been published, the dose schedule of RT/TMZ differed from the standard schedule followed in the other trials. Burri et al. included an immediate postoperative 5-day cycle of TMZ beginning on day 4 ± 1, dosed at 150–200 mg/m^2^ per day [29]. Concurrent TMZ at 75 mg/m^2^ then began at day 33 ± 1 with RT; after completion of concurrent treatment, adjuvant TMZ was begun and administered at 150–200 mg/m^2^ per day for 5 days per 28-day cycle for up to 10 cycles or until progression or patient intolerance [29]. Because the Burri et al. trial was a multisite phase II study, it was included in the analysis. Noel et al. also similarly employed 1 cycle of pre-RT TMZ in 13 of the 65 patients enrolled in their trial (including 7 of the 28 patients in the trial who were implanted with Gliadel wafers), using a dose of 150 mg/m^2^ per day for five consecutive days before RT began [34].

Altogether, in the 11 trials that met search criteria, 662 patients were treated, of whom 411 (62.1 %) with newly diagnosed HGG received Gliadel wafers followed by RT/TMZ.

### Patient characteristics

The patients analyzed in these 11 trials were relatively similar (Table 2). The majority (approximately 85 %) of these patients had newly diagnosed GBM. The mean age was 57.9 years (range 17 to 82 years), and 60.2 % of the patients were male. The presurgery performance status, measured by the Karnofsky Performance Status (KPS) scale, was ≥80 for the majority of patients. In one of the trials, the Eastern Cooperative Oncology Group (ECOG) scale was used to assess cognitive performance status; 75 % of the patients were scored either PS 0 (normal activity) or PS 1 (some symptoms) [33].

**Table 2:** Demographics and procedure characteristics reported in 11 trials of Gliadel wafers combined with standard radiotherapy and concurrent followed by adjuvant temozolomide for treatment of newly diagnosed high-grade glioma

Clinical trial	Treatments/arms	ND HGG (*n*)	Gliadel wafer plus RT/TMZ (*n*)	Mean age (range)	% male	KPS score	EOR	MGMT status	# Gliadel wafers
Aoki et al. [27]	Resection + Gliadel wafer + RT/TMZ	16	16	50 (21–63)	50.0 %	87.5 % >80	Mean 91.9 %	No	≤8
Bock et al. [28]	Resection + Gliadel wafer + RT/TMZ	44	44	57 (28–74)	63.6 %	81 ± 15.3	86 % total	No	7.3 ± 1.3
Burri et al. [29]	Resection + Gliadel wafer + early TMZ (day 4) + RT/TMZ	46	46	56 (19–73)	60.9 %	80	70 % total	Yes, performed on 22 pts	8
Duntze et al. [30]	Resection + Gliadel wafer + RT/TMZ	92	65	58 (34–76)	69.6 %	Median 80	86 % >90 %	No	6.5
McGirt et al. [31]	(1) Resection + Gliadel wafer + RT/TMZ	33	33	57 (50–81)	60.0 %	80	77 % total	No	8
	(2) Resection + Gliadel wafer + RT	78		NA			NA		
	(3) Resection/biopsy + RT/TMZ	45		(18–70)			NA		
Menei et al. [32]	(1) Resection + Gliadel wafer + RT/TMZ	43	43	60 (18–80)	58.1 %	Median 80	84.3 % >90 %	No	8
	(2) Resection + Gliadel wafer + other regimens	40							
Miglierini et al. [33]	Resection + Gliadel wafer + RT/TMZ	24	22	Mean 60.25 Median 63 5 pts >70	70.8 %	75 % PS 0–1	50 % total	No	8
Noel et al. [34]	(1) Resection + Gliadel wafer + RT/TMZ	28	28	61 (17–82)	53.6 %	92.8 % ≥80	35.7 % total	Yes	8
	(2) Resection/biopsy + RT/TMZ	37		61 (17–82)	40.5 %	81.1 % ≥80	24.3 % total		
Pan et al. [35]	Resection + Gliadel wafer + RT/TMZ	21	21	60 (48–83)	66.7 %	Median 80	67 % total	No	8
Pavlov et al. [36]	Resection + Gliadel wafer + RT/TMZ	83	61	59.9 (21–78)	60.2 %	94 % ≥70	49.1 % total	No	7.1 (3–13)
Salvati et al. [37]	Resection + Gliadel wafer + RT/TMZ	32	32	Median 58.5 (35–72)	50.0 %	Mean 80.6	100 % total	No	8 (5–10)

*EOR* extent of resection, *KPS* Karnofsky Performance Status, *ND HGG* newly diagnosed high-grade glioma, *MGMT* O^6^-methylguanine-DNA methyltransferase methylation, *RT/TMZ* radiotherapy + temozolomide regimen, *NA* not available

In the majority of these trials, the percentage of patients who underwent complete resection was near to or greater than 80 %. In three trials, however, all performed by French investigators, rates of complete resection were lower. Of the 22 patients in the study by Miglierini et al., only 50 % had complete resection [33]. Of the 38 patients in the combination-therapy arm and the 37 patients in the RT/TMZ-only arm in the study by Noel et al., only 35.7 and 24.3 %, respectively, had complete resection [34]. Of the total 83 patients in the study by Pavlov et al., 49.1 % had complete resection; 61 (73.5 %) of the 83 patients received Gliadel plus standard RT/TMZ [36].

In the study by Noel et al., which compared Gliadel plus RT/TMZ to RT/TMZ alone, there were significantly fewer patients with grade III HGG in the combination-therapy arm (*n* = 8) than in the RT/TMZ-only arm (*n* = 21) (*p* = 0.04) [34]. The investigators acknowledged that this imbalance could mask the benefit of Gliadel wafers used with RT/TMZ because of the better survival profile of grade III HGG patients compared to those with GBM.

Methylation of the promoter region of the methyl guanine methyl transferase (MGMT) gene has been demonstrated to be both a strong prognostic marker for outcome and a predictive marker for response to alkylating agents [38, 39]. Only two of the trials in this systematic review reported MGMT analysis. Burri et al. reported MGMT promoter methylation status for 22 of 43 patients with GBM (out of 46 patients total in the study) [29]. In the study by Noel et al., MGMT promoter methylation status was reported for all 65 patients: 24 patients were methylated, 27 patients were unmethylated, and 14 patients were not analyzed [34].

In these 11 clinical trial reports, there were few descriptive comments regarding procedural details for the surgical implantation of Gliadel wafers. Overall, patients received an average of eight Gliadel wafers, with a minority receiving fewer than five wafers. In the studies by Duntze and Menei, the range of Gliadel wafers implanted was reported to be from 1 to 9 [30, 32]. Duntze et al. reported no correlation between preoperative tumor volume and the number of implanted Gliadel wafers [30]. However, Pavlov et al. found a significant positive correlation between preoperative tumor volume and the number of implanted wafers (*p* < 0.001), with the number of implanted wafers increasing with tumor volume [36].

### Efficacy results

#### Median overall survival

**Fig. 2:**
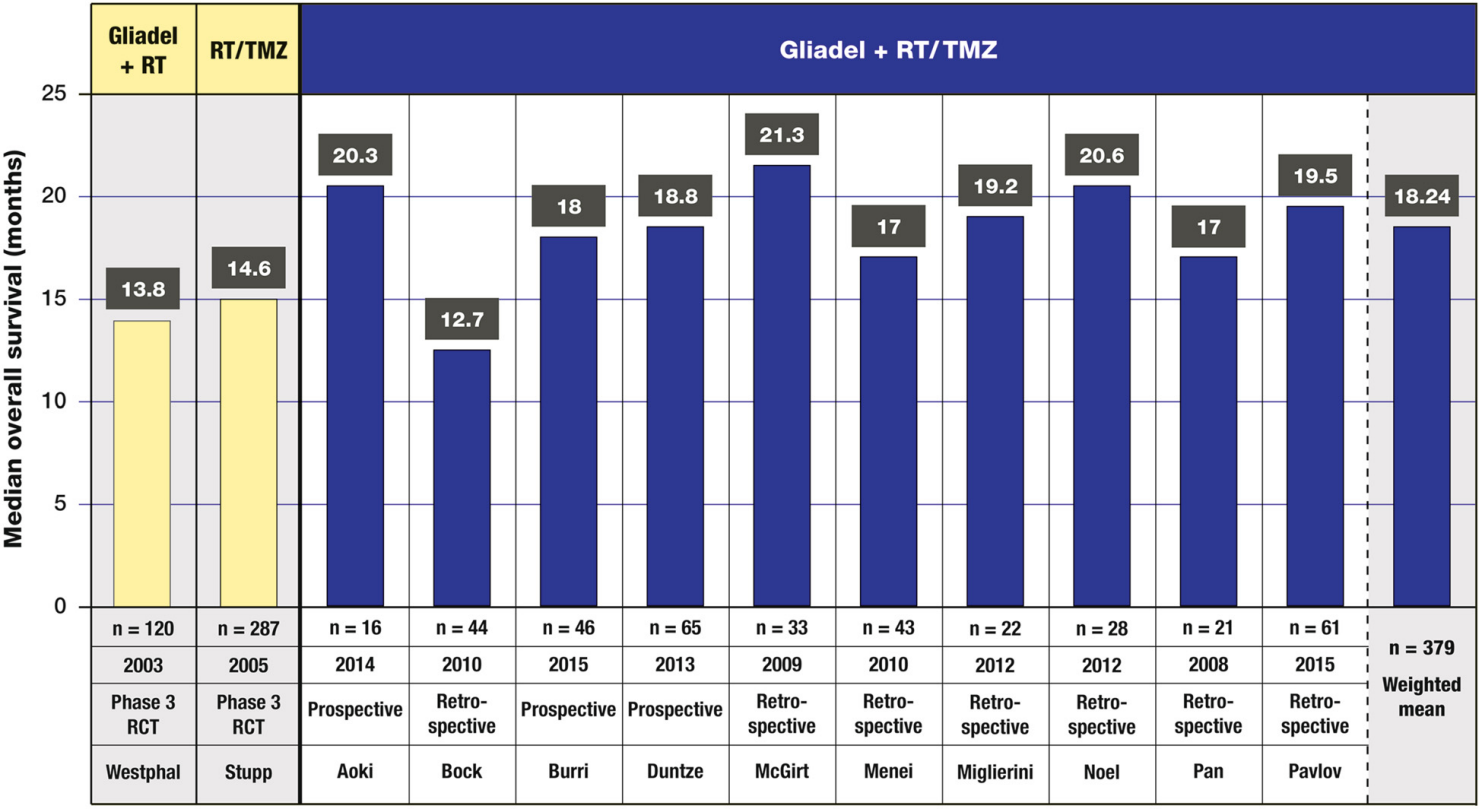
Median overall survival (OS) in months as reported in trials of Gliadel wafers combined with standard radiotherapy (RT) and concurrent followed by adjuvant temozolomide (TMZ) for the treatment of high-grade glioma. The median OS values of these trials are compared with the values for the active treatment arm of the 240-patient phase III clinical trial of Gliadel wafers plus RT [8, 10] (column 1) and the active treatment arm of the phase III clinical trial of RT/TMZ (column 2) [11, 12]

Efficacy data from these trials are presented in Table 3. The weighted mean of median OS was 18.2 months (ten trials, *n* = 379), with a range from 12.7 months (*n* = 44) to 21.3 months (*n* = 33) (Fig. 2). In the study by Mcgirt et al., median OS was longer for Gliadel plus RT/TMZ than for Gliadel wafers alone (21.3 vs 12.4 months, *p* = 0.005) or for RT/TMZ alone (21.3 vs 14.7 months, *p* < 0.001) [31]. In the study by Noel et al., median OS was not significantly different for Gliadel plus RT/TMZ compared to RT/TMZ alone (20.6 vs 20.8 months) [34]. However, this outcome for the overall study cannot be considered conclusive, because for the subset of patients with WHO grade III HGG (*n* = 29), the median OS had not been reached at the time of data analysis. For the subset of patients with GBM (*n* = 36), the median OS was reached and was demonstrably longer for Gliadel plus RT/TMZ than for RT/TMZ alone (20.8 vs 13.8 months).

**Table 3:** Efficacy data reported in 11 trials of Gliadel wafers combined with standard radiotherapy and concurrent followed by adjuvant temozolomide for treatment of newly diagnosed high-grade glioma

Clinical trial	Treatments/arms	ND HGG (*n*)	Gliadel wafer plus RT/TMZ (*n*)	Median OS (months)	OS, 1 year (%)	OS, 2 years (%)	Median PFS (months)	PFS, 6 months (%)	PFS, 1 year (%)
Aoki et al. [27]	Resection + Gliadel wafer + RT/TMZ	16	16	20.2^a^	100.0 %	68.8 %, 44.4%^a^	NA	75.0 %	62.5 %
Bock et al. [28]	Resection + Gliadel wafer + RT/TMZ	44	44	12.7	58.0 %	13.0 %	7	63.0 %	35.0 %
Burri et al. [29]	Resection + Gliadel wafer + early TMZ (day 4) + RT/TMZ	46	46	18	76.0 %	33.0 %	8.5	72.0 %	33.0 %
Duntze et al. [30]	Resection + Gliadel wafer + RT/TMZ	92	65	18.8	70.0 %	37.0 %	10.5	74 %	41.0 %
McGirt et al. [31]	(1) Resection + Gliadel wafer + RT/TMZ	33	33	21.3	NA	36.0 %	NA	93.0 %	NA
	(2) Resection + Gliadel wafer + RT	78		12.4^b^	NA	NA	NA	NA	NA
	(3) Resection/biopsy + RT/TMZ	45		14.7^c^	NA	NA	NA	NA	NA
Menei et al. [32]	(1) Resection + Gliadel wafer + RT/TMZ	43	43	17	NA	NA	NA	NA	NA
	(2) Resection + Gliadel wafer + other regimens	40		NA	NA	NA	NA	NA	NA
Miglierini et al. [33]	Resection + Gliadel wafer + RT/TMZ	24	22	19.2	78.0 %	24.0 %	12.3	81.50 %	52.0 %
Noel et al. [34]	(1) Resection + Gliadel wafer + RT/TMZ	28	28	20.6	78.6 %	40.9 %	12.9	NA	52.0 %
	(2) Resection/biopsy + RT/TMZ	37		20.8	78.4%^d^	33.3 %	14	NA	55.0%^e^
Pan et al. [35]	Resection + Gliadel wafer + RT/TMZ	21	21	17	NA	39.0 %	8.5	71.0 %	NA
Pavlov et al. [36]	Resection + Gliadel wafer + RT/TMZ	83	61	19.5	NA	NA	8.5	NA	NA
Salvati et al. [37]	Resection + Gliadel wafer + RT/TMZ	32	32	NA	100 %	NA	NA	100 %	NA

*EOR* extent of resection, *ND HGG* newly diagnosed high-grade glioma, *OS* overall survival, *PFS* progression-free survival, *RT/TMZ* radiotherapy + temozolomide regimen, *NA* not available

^a^Glioblastoma patients only

^b^Gliadel plus RT/TMZ versus Gliadel wafer alone, *p* = 0.005

^c^Gliadel plus RT/TMZ versus RT/TMZ, *p* < 0.001

^d^Gliadel plus RT/TMZ versus RT/TMZ, log-rank test for overall survival, *p* = 0.81

^e^Gliadel plus RT/TMZ versus RT/TMZ, log-rank test for progression-free survival, *p* = 0.89

#### Overall 1- and 2-year survival rates

**Fig. 3:**
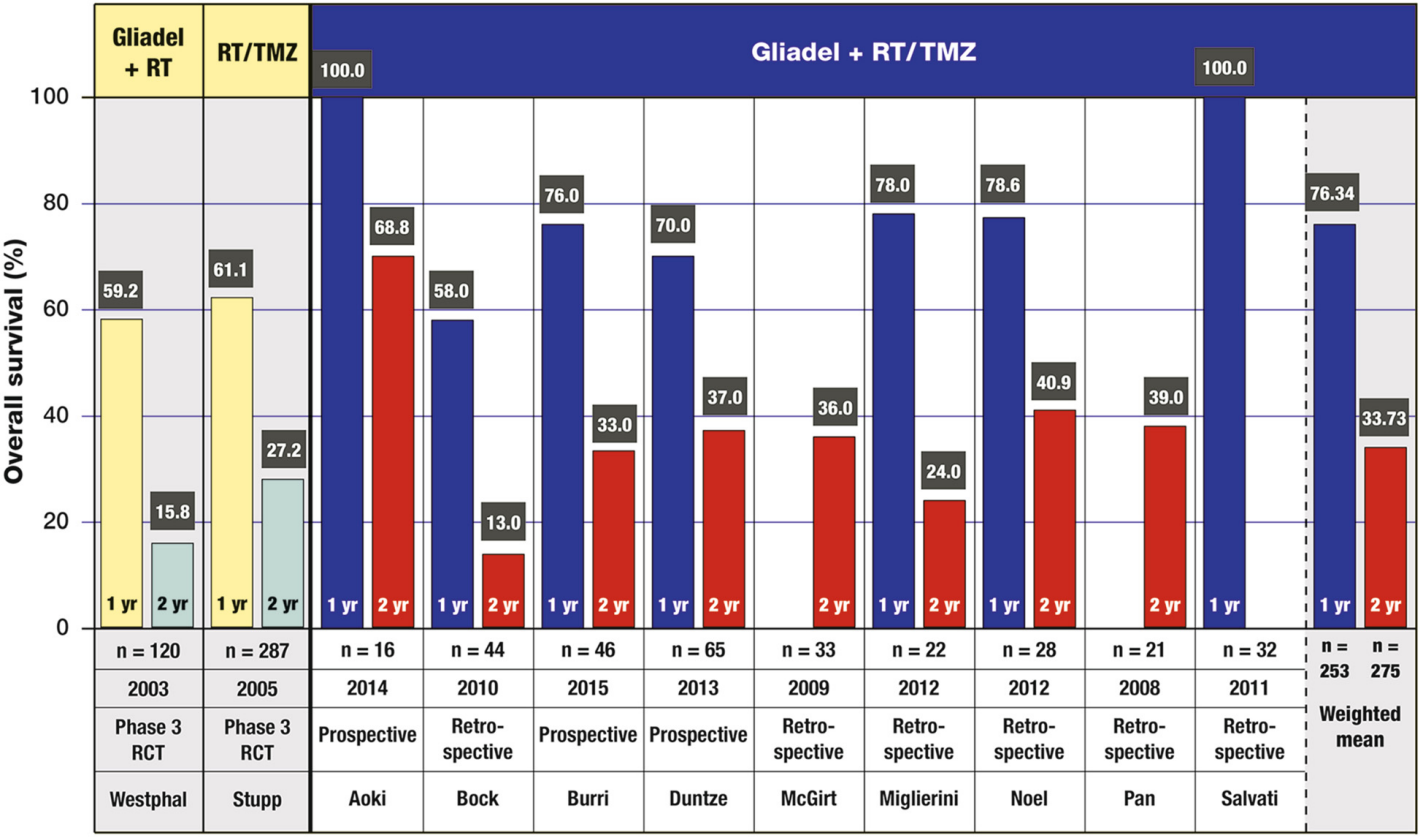
Overall survival (OS) at 1 year and 2 years as reported in trials of Gliadel wafers combined with standard radiotherapy (RT) and concurrent followed by adjuvant temozolomide (TMZ) for the treatment of high-grade glioma. The 1-year and 2-year OS values of these trials are compared with the values for the active treatment arm of the 240-patient phase III clinical trial of Gliadel wafers plus RT [8, 10] (column 1) and the active treatment arm of the phase III clinical trial of RT/TMZ (column 2) [11, 12]

The weighted mean of 1-year OS rates in these trials was 76.34 % (seven trials, *n* = 253), with a range of 58.0 % (*n* = 44) to 100 % (*n* = 16; *n* = 32); and the weighted mean of 2-year OS rates was 33.73 % (eight trials, *n* = 275), with a range of 13.0 % (*n* = 44) to 68.8 % (*n* = 16) (Fig. 3). In the study by Noel et al., there was no significant difference in 1-year survival rates between Gliadel plus RT/TMZ and RT/TMZ alone (78.6 vs 78.44 %, respectively, log-rank *p* = 0.89) [34]. For the subset of patients with GBM, the differences in 1-year and 2-year survival rates between Gliadel plus RT/TMZ therapy and RT/TMZ alone were 75.0 versus 62.5 %, and 38.9 versus 0 %, respectively, approaching statistical significance (log-rank *p* = 0.067).

#### Progression-free survival

**Fig. 4:**
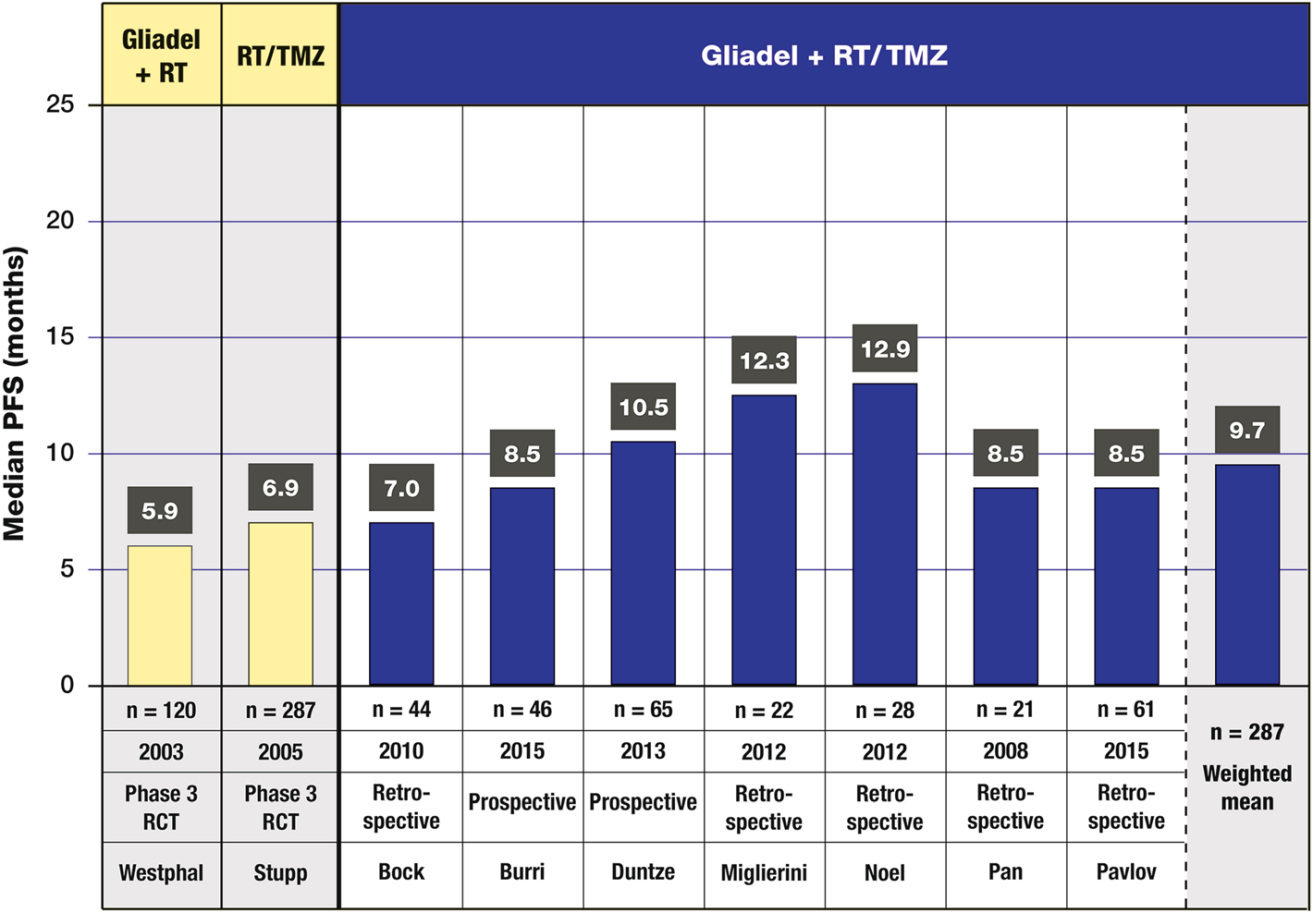
Median progression-free survival (PFS) in months as reported in trials of Gliadel wafers combined with standard radiotherapy (RT) and concurrent followed by adjuvant temozolomide (TMZ) for the treatment of high-grade glioma. The median PFS values of these trials are compared with the values for the active treatment arm of the 240-patient phase III clinical trial of Gliadel wafers plus RT [8, 10] (column 1) and the active treatment arm of the phase III clinical trial of RT/TMZ (column 2) [11, 12]

The weighted mean of median PFS in these trials was 9.7 months (seven trials, *n* = 287), with a range from 7 months (*n* = 44) to 12.9 months (*n* = 28) (Fig. 4). The weighted mean for PFS was 78.7 % at 6 months (eight trials, *n* = 279) and 45.9 % at 12 months (six trials, *n* = 221). PFS ranged from 63.0 % (*n* = 44) to 100 % (*n* = 32) at 6 months and from 33.0 % (*n* = 46) to 62.5 % (*n* = 16) at 12 months. In the study by Noel et al., median PFS was lower for Gliadel plus RT/TMZ than for RT/TMZ alone (12.9 vs 14 months), and the PFS rate at 12 months (no 6-month rates were provided) for Gliadel plus RT/TMZ was also less than that for RT/TMZ alone (52.0 vs 55.0 %), but these rates were not significantly different (log-rank *p* = 0.89) [34]. In contrast, for the subset of patients in the Noel et al.’s trial with GBM, the median PFS was greater for Gliadel plus RT/TMZ than for RT/TMZ alone (9.7 vs 7.8 months), as were the PFS rates at 6 and 12 months (6 months, 73.7 vs 64.6 %; 12 months, 36.8 vs 32.3 %); however, these PFS rates were again not significantly different (log-rank *p* = 0.4).

#### Subgroup analyses and prognostic factors

In patients treated with Gliadel plus RT/TMZ, Duntze et al. found no statistically significant difference in OS associated with tumor grade, KPS score ≥70, or age [30]. However, these investigators did find a difference approaching statistical significance for PFS between patients with <90 % resection and patients with ≥90 % resection (*p* = 0.057).

By multivariate analysis, Noel et al. found that unmethylated MGMT was a negative prognostic factor for PFS (*p* = 0.017, HR 2.8, 95 % CI 1.2–7) and that the combination of unmethylated MGMT and a radiation dose <60 Gy was a negative prognostic factor for OS (*p* = 0.02, HR 6.3, 95 % CI 2–20) [34]. In the Burri et al. clinical trial, patients with unmethylated MGMT (*n* = 19) experienced shorter median OS but longer median PFS than patients with methylated MGMT (*n* = 14) (18.2 vs 27.3 months for median OS; 8.9 vs 7.4 months for median PFS) [29].

By multivariate analysis, Pavlov et al. found that tumor volume ≥40 cm^3^ (HR 3.62, 95 % CI 1.53–9.05, *p* = 0.003), subtotal or total resection (HR 0.45, 95 % CI 0.21–0.99, *p* = 0.005), and ≥8 Gliadel wafers (HR 0.32, 95 % CI 0.14–0.74, *p* = 0.008) were positive prognostic factors for OS in patients who received Gliadel plus RT/TMZ (*n* = 61) [36]. Patients implanted with ≥8 Gliadel wafers survived significantly longer than patients implanted with <8 Gliadel wafers (median OS 24.5 vs 13 months, *p* = 0.021). Patients who had ≥6 cycles of adjuvant TMZ survived significantly longer than patients who had <6 cycles of adjuvant TMZ (27 vs 16 months, *p* < 0.001).

### Safety results

Grade 3 and 4 adverse events were reported in nine of the 11 trials and are categorized and listed in Table 4. The trials by McGirt and Menei did not specifically designate the severity of adverse events using *CTCAE* adverse-event grades [31, 32]. Also, two of the trials did not break out adverse events for the subgroup of patients with newly diagnosed HGG who specifically received Gliadel plus RT/TMZ. Aoki et al. merged adverse events for 16 patients receiving Gliadel plus RT/TMZ for newly diagnosed HGG with those for eight patients receiving Gliadel wafers for recurrent GBM [27]. Duntze et al. reported merged adverse events for all 92 patients with newly diagnosed HGG who received Gliadel wafers—a population that included but was not limited to the 65 patients in that study who specifically received Gliadel wafers plus RT/TMZ [30].

**Table 4:** Grade 3 and 4 adverse events reported in nine trials of Gliadel wafers combined with standard radiotherapy and concurrent followed by adjuvant temozolomide for treatment of newly diagnosed high-grade glioma

Clinical trial	Number	CE	HA	IH	II	ND	S	LE	F	GI	MY	SK	TE	Total, *n*
Aoki et al. [27]^a^	24	2				5	1	2	1		4			15
Bock et al. [28]	44	7	11		6	8	7				1		6	46
Burri et al. [29]	46	1	2	7	3		3	3			18		2	39
Duntze et al. [30]^b^	92		3	2	5	12					7			29
Miglierini et al. [33]	24				3						3			6
Noel et al. [34]	28										4			4
Pan et al. [35]	21				1	1								2
Pavlov et al. [36]	61			2		3					1			6
Salvati et al. [37]	32		2											0
Total, *n*	372	10	16	11	18	29	11	5	1	1	38	1	8	147
Total, %		2.7 %	4.3 %	3.0 %	4.8 %	7.8 %	3.0 %	1.3 %	0.3 %	0.3 %	10.2 %	0.2 %	2.15 %	39.5 %

*CE* cerebral edema, *HA* healing abnormalities (including CSF leak and hydrocephalus), *IH* intracranial hypertension, *II* intracranial infections (including abscess, cerebritis, hydrocephitis, and meningitis), *ND* neurological deficit (including aphasia, change of mental status, epilepsy, and hemiparesis), *S* seizures, *LE* elevated liver enzymes, *F* fatigue (including fever), *GI* gastrointestinal disorders (including nausea, vomiting, and constipation), *MY* myelosuppression (including anemia, lymphopenia, leukocytopenia, neutropenia, and thrombocytopenia), *SK* skin and subcutaneous disorders, *TE* thromboembolic events (including deep vein thrombosis and pulmonary embolism)

^a^Includes 16 patients treated for newly diagnosed high-grade glioma and 8 patients treated for recurrent high-grade glioma

^b^Includes all 92 patients treated with Gliadel wafers, of whom 65 received Gliadel plus RT/TMZ

In the nine trials that reported grade 3 and 4 adverse events, 147 grade 3 and 4 adverse events were registered for 372 patients. The most commonly reported grade 3 and 4 adverse events were myelosuppression (10.22 %, *n* = 38), neurological deficit (7.8 %, *n* = 29), healing abnormalities (4.3 %, *n* = 16), and seizures (3.0 %, *n* = 11).

Two of the trials reported a considerable majority of the grade 3 and 4 adverse events. In the retrospective analysis by Bock et al., 19 patients (43 %) experienced 46 grade 3 and 4 adverse events [28]. These investigators found a higher incidence of intracranial infections and CSF leaks than in the original phase III clinical trials of Gliadel wafers, some of these adverse events occurring after discharge. However, Bock et al. noted that after introducing a risk-management strategy to address patient selection, surgical techniques, and follow-up schedules, the incidence of implantation-site-related complications dropped significantly [40]. In the phase II clinical trial by Burri et al., in which 39 adverse events were reported, adjuvant TMZ was administered at a dose of 150–200 mg/m^2^ [29]. The investigators reported that nine of the patients receiving 200 mg/m^2^ of TMZ experienced one or more episodes of grade 3 or 4 thrombocytopenia after the initial pre-radiotherapy cycle of adjuvant TMZ.

Except for the one trial reported by Bock et al. [28], none of the others found that combining Gliadel plus RT/TMZ caused more myelosuppression or other adverse events than either Gliadel wafers alone or RT/TMZ alone [31]. As expressly noted by Duntze et al. regarding the results of their prospective clinical trial, there was no apparent enhancement of toxicity due to the combination of Gliadel plus RT/TMZ [30].

## Discussion

### Efficacy of Gliadel plus RT/TMZ

This systematic literature review identified 11 clinical trials in which Gliadel wafers were used in combination with RT/TMZ. Of these, eight were retrospective and three were prospective clinical trials. Altogether, in these trials, 411 patients with newly diagnosed HGG received Gliadel plus RT/TMZ.

Systematic review of the efficacy data of these 11 trials strongly suggests that a therapeutic benefit is achieved when combining Gliadel wafers and RT/TMZ. The weighted mean of median OS was 18.2 months (ten trials, *n* = 379, range 12.7 to 21.3 months), and the weighted mean of PFS was 9.7 months (seven trials, *n* = 287, range 7 to 12.9 months). These outcomes are better than those seen in the treatment arms of the original phase III clinical trials in which Gliadel wafers or TMZ were used alone with RT. In the 240-patient phase III clinical trial of Gliadel wafers, in which patients were randomized to undergo surgical resection with active or placebo wafer placement, followed by RT, median OS was improved from 11.6 to 13.9 months [8]. In both arms of that trial, median PFS was 5.9 months based on radiographic and clinical criteria. In the 573-patient EORTC/NCIC phase III study of RT/TMZ, in which patients were randomized to RT alone or RT with concomitant TMZ followed by 6 cycles of adjuvant TMZ, median OS was improved from 12.1 to 14.6 months, and median PFS was improved from 5.0 to 6.9 months [11]. The difference in median OS between the 11 trials analyzed in the current review and the treatment arm of the EORTC/NCIC RT/TMZ trial (18.2 vs 14.6 months, a difference of 3.6 months) exceeds the difference in median OS between the RT/TMZ and RT-alone arms in the EORTC/NCIC clinical trial (14.6 vs 12.1 months, a difference of 2.5 months).

Pallud et al. retrospectively conducted multivariate and case-matched analyses (controlled propensity-matched cohorts) comparing outcomes for patients who received Gliadel plus RT/TMZ (*n* = 354) versus patients who received RT/TMZ alone (*n* = 433) [41]. The French multicenter cohort that comprised their analysis incorporated patients from several of the trials of Gliadel plus RT/TMZ included in the current systematic review [30, 36]. Median OS and PFS were 20.4 months (95 % CI 19.0–22.7) and 12.0 months (95 % CI 10.7–12.6) for Gliadel plus RT/TMZ versus 18.0 months (95 % CI 17.0–19.0) and 10.0 months (95 % CI 9.0–10.0) for RT/TMZ alone (*p* = 0.0048). For Gliadel plus RT/TMZ versus RT/TMZ alone, 1-year OS rates were 80.8 % (95 % CI 76.3–84.6) versus 71.3 % (95 % CI 67.0–75.2), and 2-year OS rates were 41.0 % (95 % CI 35.2–47.2) versus 30.4 % (95 % CI 26.2–34.9). The combination of Gliadel plus standard RT/TMZ was independently associated with longer PFS in patients with subtotal/total surgical resection in the whole series (adjusted HR 0.76, 95 % CI 0.63–0.92, *p* = 0.005) and after propensity matching (HR 0.74, 95 % CI 0.60–0.92, *p* = 0.008). No survival benefit was found for Gliadel plus RT/TMZ for partial resection. Gliadel plus RT/TMZ was not independently associated with longer OS in the whole series analysis (HR 0.95, 95 % CI 0.80–1.13, *p* = 0.561) or after propensity matching (HR 1.06, 95 % CI 0.87–1.29, *p* = 0.561). The investigators observed that one explanation for the finding that Gliadel plus RT/TMZ was not an independent predictor of OS was the use of salvage therapies after progression—including salvage implantation of Gliadel wafers in 20.1 % of patients who had received RT/TMZ alone but in only 4.3 % of patients who had already received Gliadel wafers.

The positive findings of our systematic literature review, suggesting additional months of survival with Gliadel plus RT/TMZ, could constitute an artifact of temporal bias, reflecting improved surgical procedures and more aggressive current neuro-oncologic care compared with the experience of historical controls and/or patient selection bias. On the other hand, the increased survival benefit could be easily explained by the antineoplastic effect of the Gliadel wafers while awaiting the start of RT/TMZ.

TMZ has demonstrated the ability to deplete the MGMT repair protein levels in tumor cells [38], resulting in improved efficacy of alkylating agents, which may contribute to the therapeutic synergy seen when combining this agent with Gliadel. Lechapt-Zalcman et al. retrospectively analyzed 111 tumors from the patients in the prospective trial conducted by Duntze et al. to determine the prognostic impact of MGMT on those treated with Gliadel plus RT/TMZ [42]. In the whole patient group, median OS was 17.5 months and median PFS was 10.3 months. Patients with methylated MGMT had significantly longer median OS than patients with unmethylated MGMT (21.7 vs 15.1 months, *p* = 0.025). Smith et al. reported a positive correlation of MGMT promoter methylation with survival in a prospective trial of 30 newly diagnosed GBM patients treated with Gliadel wafers and Gamma Knife radiosurgical boost followed by standard fractionated RT (60 Gy over 6 weeks) (but without TMZ, for the EORTC/NCIC data had not yet been published) [43]. Among the 11 trials identified for the current analysis, only two undertook MGMT testing, and none assessed any other relevant biomarkers.

### Safety review

Combination therapy regimens expose patients not only to the adverse events associated with individual treatments but also to the additive and potentially synergistic adverse effects that might derive from the combination of treatments. In the nine trials that reported grade 3 and 4 adverse events, representing 372 patients who received Gliadel plus RT/TMZ, there were 147 grade 3 and 4 adverse events—the most common being myelosuppression, neurologic deficit, healing abnormalities, and seizures. No increased incidence of radiation necrosis has been reported in this patient population, despite the use of RT following the implantation of Gliadel wafers. This is possibly due to the delay of 3 to 4 weeks that naturally occurs before the start of contemporary RT. Another possible explanation for the lack of increase in local toxicity associated with the combination of Gliadel and RT is that radiation necrosis usually occurs many months post-RT, peaking at approximately 18 months in a population of patients with a constant attrition due to progression of their disease.

To date, no large prospective controlled trial has documented the adverse-event profile of the combination of Gliadel wafers with RT/TMZ. However, the adverse events associated with the individual components of this multimodal regimen have been prospectively studied: for Gliadel wafers with postoperative external beam RT (60 Gy) in the phase III trials reported by Valtonen et al. [9] and Westphal et al. [8, 10] and for TMZ used concurrently with and then following 60-Gy involved field cranial radiation in the EORTC/NCIC phase III trial reported by Stupp et al. [11]. The adverse events in these phase III trials are listed in Table 5.

**Table 5:** Adverse events associated with Gliadel wafers combined with standard radiotherapy (RT) and concurrent followed by adjuvant temozolomide (TMZ) compared with adverse events in (1) the phase III RT/TMZ clinical trial, (2) the phase III Gliadel clinical trials, and (3) the Attenello et al.’s retrospective comparison of patients who underwent craniotomy with and without Gliadel wafer implantation

Clinical trial	Number	CE	HA	IH	II	ND	S	LE	F	GI	MY	SK	TE	UI
Grade 3 and 4 AEs, 9 Gliadel wafers + RT/TMZ, %	372	2.7 %	4.3 %	3.0 %	4.8 %	7.8 %	3.0 %	1.3 %	0.3 %	0.3 %	10.2 %	0.2 %	2.15 %	
Grade 3 and 4 AEs, Phase III RT/TMZ trial, [11, 12] RT/TMZ treatment arm, %	287				7.0 %				13.0 %	2.0 %	16.0 %	3.0 %		6.0 %
AEs, Gliadel phase III clinical trials, Gliadel wafer arm, % [8–10, 25]	120	23.0 %	16.0 %	9.0 %	5.0 %	16.0 %	33.0 %		27.0 %	700 %			18.0 %	
AEs, Gliadel phase III clinical trials, placebo wafer arm, % [8–10, 25]	120	19.0 %	12.0 %	2.0 %	6.0 %	10.0 %	38.0 %		15.0 %	47.0 %			17.0 %	
AEs, Attenello et al., [46] Gliadel wafer arm, %	288	2.3 %	2.8 %		1.2 %		14.6 %				0.3 %		11.2 %	
AEs, Attenello et al., [46] placebo arm, %	725	2.1 %	2.2 %		0.7 %		15.7 %				0.3 %		8.9 %	

*AE* adverse events, *CE* cerebral edema, *HA* healing abnormalities (including CSF leak and hydrocephalus), *IH* intracranial hypertension, *II* intracranial infections (including abscess, cerebritis, hydrocephitis, and meningitis), *ND* neurological deficit (including aphasia, change of mental status, epilepsy, and hemiparesis), *S* seizures, *LE* elevated liver enzymes, *F* fatigue (including fever), *GI* gastrointestinal disorders (including nausea, vomiting, and constipation), *MY* myelosuppression (including anemia, lymphopenia, leukocytopenia, neutropenia, and thrombocytopenia), *SK* skin and subcutaneous disorders, *TE* thromboembolic events (including deep vein thrombosis and pulmonary embolism), *UI* unidentified

In the nine trials reporting grade 3 and 4 adverse events, these events seemed to reflect the distinct and independent adverse-event profiles of Gliadel wafers and RT/TMZ, considered separately. Specifically, there was little evidence of any enhanced toxicity from combination therapy. Carmustine, when released from the polymeric wafers into local tissue after implantation, results in only trace amounts of drug in the systemic circulation. This suggests that adverse events such as fatigue, gastrointestinal disorders, and myelosuppression are likely due to the systemic toxicity of RT/TMZ without influence from the local effects of the Gliadel wafer implant [44].

In the phase III clinical trials of Gliadel wafers, adverse events were not graded, but there was an explicit comparison between the active and placebo treatment arms (Table 5) [8, 10, 25]. Neurological adverse events in the treatment arms—seizures, neurological deficits, and operative complications—were similar. Further, postoperative complications were similar, except for (1) healing abnormalities commonly related to CSF leaks (16.0 % for the active treatment arm vs 12.0 % for placebo) and (2) intracranial hypertension (9.0 vs 2.0 %). Since the FDA approval of Gliadel wafers for newly diagnosed HGG in 2003, risk-management articles have recommended “best-practice” surgical techniques for reducing adverse events associated with Gliadel wafer implantation [40, 45]. In this regard, ensuring a watertight dural seal is mandatory for reducing any risk of CSF leak.

That the adverse-event profile of Gliadel wafers can be safely managed with good technique is evidenced by reports from the Johns Hopkins University School of Medicine, where the Gliadel wafer technology was originally developed. In 2008, Attenello et al. reported institutional experience in 288 HGG patients (166 newly diagnosed, 122 recurrent) implanted with Gliadel wafers compared with 725 patients who underwent craniotomy without Gliadel implants [46]. For the patients implanted with Gliadel wafers versus the patients who underwent craniotomy without wafers, Attenello et al. found similar incidences of perioperative infection at the surgical site (2.8 vs 1.8 %, *p* = 0.33), CSF leakage (2.8 vs 1.8 %, *p* = 0.33), meningitis (0.3 vs 0.3 %, *p* = 1.00), incisional wound healing difficulty (0.7 vs 0.4 %, *p* = 0.63), symptomatic malignant edema (2.1 vs 2.3 %, *p* = 1.00), seizures at 3 months (14.6 vs 15.7 %, *p* = 0.65), DVT (6.3 vs 5.2 %, *p* = 0.53), and pulmonary embolism (4.9 vs 3.7 %, *p* = 0.41) (Table 5).

More recently, Chaichana et al. from the Johns Hopkins School of Medicine retrospectively analyzed all patients who had undergone resection of GBM from 2007 to 2011, using multivariate proportional hazards regression analysis to identify factors associated with infection, including Gliadel wafers [47]. During that time, 401 patients underwent resection, of whom 21 (5 %) developed infection at a median 40 days after surgery. The incidence of infection was not higher in patients who received Gliadel wafers.

## Conclusions

This systematic literature analysis was conducted in the context of renewed interest in Gliadel wafers following the disappointing results from recent phase III clinical trials of other therapies for GBM. In the last two decades, Gliadel wafers and TMZ have been the only chemotherapy agents for the treatment of newly diagnosed GBM that have been confirmed by phase III randomized clinical trials and subsequently approved by the FDA, and only Gliadel wafers have been approved for treatment of WHO grade III gliomas.

The rationale for polymeric delivery systems for treatment of HGG, and with that the history of Gliadel wafers, has been extensively reviewed [44]. Moreover, two meta-analyses have recently affirmed the survival benefit associated with Gliadel wafers. In one of these meta-analyses, Xing et al. examined six randomized controlled studies and four cohort studies for the treatment of newly diagnosed HGG [48]. In the other of these meta-analyses, Chowdhary et al. examined 40 studies and 22 abstracts for the treatment of both newly diagnosed HGG and recurrent GBM [49].

Our findings suggest that when Gliadel wafers and RT/TMZ are combined, there is a positive additive effect of improving survival without an increase in toxicity. The survival benefit appears to be greater than that seen individually for the separate treatments in their respective phase III studies compared to placebo wafers with RT [8–10] or to RT alone [11]. Because improved survival outcomes can be offered to HGG patients with agents that are FDA-approved and recommended by guidelines [14, 15, 50], these therapies should be applied to maximal advantage when access is not available to a well-designed rational clinical trial. Moreover, exposure to these local therapies should not exclude patients from clinical trial enrollment but should instead be used as stratification factors for subgroup analyses.

Larger prospective trials of Gliadel plus RT/TMZ are required for definitive prospective analysis of efficacy and safety and identification of patients who might benefit most from this sequential combination of treatments. Our review is hypothesis-generating and supports conducting a phase III clinical trial to compare the RT/TMZ regimen alone versus the multimodal Gliadel plus RT/TMZ regimen. In the meantime, two large prospective registries now underway involving the use of Gliadel wafers for newly diagnosed HGG—one in Japan with 250 patients (NCT02300506), the other in the USA enrolling 500 patients—could confirm and extend the findings reported in the current systematic review.

## Abbreviations

AE: Adverse event
CI: Confidence interval
CSF: Cerebral spinal fluid
CTCAE: Common Terminology Criteria for Adverse Events
ECOG: Eastern Cooperative Oncology Group
EORTC: European Organization for Research and Treatment of Cancer
FDA: US Food and Drug Administration
GBM: Glioblastoma multiforme
HGG: High-grade glioma
HR: Hazard ratio
KPS: Karnofsky Performance Status
MGMT: Methyl guanine methyl transferase
NCIC: National Cancer Institute of Canada
OS: Overall survival
PFS: Progression-free survival
RT: Radiotherapy
TMZ: Temozolomide
WHO: World Health Organization
